# Role of Native Defects in Fe-Doped β-Ga_2_O_3_

**DOI:** 10.3390/ma16206758

**Published:** 2023-10-19

**Authors:** Hui Zeng, Meng Wu, Haixia Gao, Yuansheng Wang, Hongfei Xu, Meijuan Cheng, Qiubao Lin

**Affiliations:** 1 College of Science, Hunan University of Science and Engineering, Yongzhou 425199, China; 2 College of Materials Science and Engineering, Hunan University, Changsha 410082, China; 3 Fujian Provincial Key Laboratory of Semiconductors and Applications, Collaborative Innovation Center for Optoelectronic Semiconductors and Efficient Devices, Department of Physics, Xiamen University, Xiamen 361005, China; 4 College of Science, Jimei University, Xiamen 361021, China

**Keywords:** first-principles, β-Ga_2_O_3_, doping, impurity levels, defect formation energies, optical properties

## Abstract

Iron impurities are believed to act as deep acceptors that can compensate for the n-type conductivity in as-grown Ga_2_O_3_, but several scientific issues, such as the site occupation of the Fe heteroatom and the complexes of Fe-doped β-Ga_2_O_3_ with native defects, are still lacking. In this paper, based on first-principle density functional theory calculations with the generalized gradient approximation approach, the controversy regarding the preferential Fe incorporation on the Ga site in the β-Ga_2_O_3_ crystal has been addressed, and our result demonstrates that Fe dopant is energetically favored on the octahedrally coordinated Ga site. The structural stabilities are confirmed by the formation energy calculations, the phonon dispersion relationships, and the strain-dependent analyses. The thermodynamic transition level Fe^3+^/Fe^2+^ is located at 0.52 eV below the conduction band minimum, which is consistent with Ingebrigtsen’s theoretical conclusion, but slightly smaller than some experimental values between 0.78 eV and 1.2 eV. In order to provide direct guidance for material synthesis and property design in Fe-doped β-Ga_2_O_3_, the defect formation energies, charge transitional levels, and optical properties of the defective complexes with different kinds of native defects are investigated. Our results show that V_Ga_ and O_i_ can be easily formed for the Fe-doped β-Ga_2_O_3_ crystals under O-rich conditions, where the +3 charge state Fe_Ga_Ga_i_ and −2 charge state Fe_Ga_O_i_ are energetically favorable when the Fermi level approaches the valence and conduction band edges, respectively. Optical absorption shows that the complexes of Fe_Ga_Ga_i_ and Fe_Ga_V_Ga_ can significantly enhance the optical absorption in the visible-infrared region, while the energy-loss function in the β-Ga_2_O_3_ material is almost negligible after the extra introduction of various intrinsic defects.

## 1. Introduction

Gallium oxides (Ga_2_O_3_) have received a lot of attention due to their exceptional physical and chemical features with a variety of applications such as solar-blind ultraviolet photodetectors [1,2], high-power transistors [3,4], Schottky diodes [5,6], as well as photocatalysts [7]. Due to the inevitable insertion of native defects (such as Ga_i_ [8,9]) and extrinsic impurities (such as Si [10], H [11]) during the growth of materials, perfect Ga_2_O_3_ exhibits n-type conductivity, which severely impedes its further applications. Doping engineering, in general, can be a valuable approach to manipulating conductivity, which influences electrical and optical performance by modifying the microscopic crystalline structure [12,13,14,15,16]. As a result, studies of acceptors in β-Ga_2_O_3_ materials are required. The n-type conductivity in perfect β-Ga_2_O_3_ can be compensated by the introduction of deep acceptors, such as Fe dopant. Fe impurity is one of the most attractive dopants because it not only exists unintentionally during the synthesis of β-Ga_2_O_3_ crystals but also possesses the same tri-valence states with comparable ionic radii as host Ga^3+^ [17].

β-Ga_2_O_3_ exhibits a monoclinic structure with two nonequivalent tetrahedrally and octahedrally coordinated Ga sites. Regarding the replacement of Ga by Fe dopant in the β-Ga_2_O_3_ system, one general question would be what is the preferential substitution location for Fe, the tetrahedrally or the octahedrally coordinated Ga sites? Previous studies showed that the specified site location and local symmetry may determine the microscopic structure as well as the optical and electronic properties [18]. Zhang et al. reported the preferential occupation of Fe^3+^ ions in the octahedral over the tetrahedral sites in the Fe-doped β-Ga_2_O_3_ crystal based on electron paramagnetic resonance (EPR) analyses [19]. Trooster et al. reported that Fe-doped Ga_2-x_Fe_x_O_3_ powder with high doping concentrations (i.e., x = 0.8, 0.9, 1.08, and 1.15) grown with the flux method of Remeika exhibited ferrimagnetic spin configuration. Based on the Mossbauer measurements of ^57^Fe in different compositions, they concluded that Fe^3+^ ions mainly replaced Ga^3+^ in the octahedral sites, with only a small fraction of Fe ions at the tetrahedral Ga sites [20]. Büscher et al. revealed the occupation of Fe^3+^ in distorted tetrahedral sites in single β-Ga_2_O_3_ crystals for the first time by employing EPR measurements [21]. Recently, Bhandari et al. showed that the Fe-replaced tetrahedral and octahedral Ga sites were not distinguished since the photon-induced changes at two different Ga sites were the same based on steady-state photo-EPR measurements [22]. This controversy over the specified Fe location in β-Ga_2_O_3_ will be addressed theoretically as below. In addition, native defects are inevitable in Fe-doping in the β-Ga_2_O_3_ system. Zhang et al. revealed that, different from the as-grown Fe-doped β-Ga_2_O_3_ system, the air-annealing treatment can efficiently increase the crystalline quality and reduce oxygen vacancies, along with decreasing the conductivity and halving the spin susceptibility [19]. Zhou et al. found that for the Fe substituting of the Ga site in the β-Ga_2_O_3_ lattice, the main defects originated from oxygen vacancies at room temperatures, as suggested by the EPR spectra, which led to the high resistivity and the potential application for *x*-ray detection [23]. Hany et al. also reported the existence of V_O_ and V_Ga_ in Fe-doped β-Ga_2_O_3_ single crystals using optical absorption and temperature-dependent cathodoluminescence (CL) measurements [24].

In terms of the electronic structure variation due to Fe^3+^ substitution, a deep level between (0.78–1.2) eV relative to the conduction band minimum (CBM) has been reported. Ingebrigtsen et al. unfolded two similar deep levels located at 0.78 eV and 0.75 eV, which can be associated with Fe impurities and intrinsic defects, respectively, for both bulk β-Ga_2_O_3_ crystal growth by hydride vapor phase epitaxy and molecular beam epitaxy methods [25]. Lenyk et al. studied the Fe^3+^/Fe^2+^ level in Fe-doped β-Ga_2_O_3_ crystals and observed a value of ~0.84 eV below the conduction band using noncontact spectroscopy methods including EPR, infrared absorption, and thermoluminescence [17]. Polyakov et al. ascertained that the Fermi level (E_f_) in Fe-doped β-Ga_2_O_3_ crystals was pinned by the Fe acceptor level near the CBM of ~0.80 eV [26]. Bhandari et al. showed that Fe dopants act as deep acceptors, and the first optically induced change in Fe^3+^ occurred at 1.2 eV in a Fe-doped β-Ga_2_O_3_ single crystal [22].

To corroborate the experimentally resolved two similar deep levels, Ingebrigtsen et al. performed Heyd-Scuseria-Ernzerhof (HSE) hybrid functional calculations. Their results show that the Fe-replaced Ga site is energetically favored on the octahedral Ga site (Fe_GaII_), while Fe substituted for the tetrahedral site (Fe_GaI_) exhibits a slightly higher formation energy both under O-rich and Ga-rich conditions compared with the Fe_GaII_ state. Moreover, the thermodynamic transition level for the Fe^3+^/Fe^2+^ is located at 0.61 eV below the CBM; the level falls to 0.40 eV if assuming the lower screening [25]. We can hardly find more theoretically calculated results for Fe-doped β-Ga_2_O_3_ to the best of our knowledge. Therefore, it is highly desirable to provide a fundamental understanding of the relationships between the local crystal structure in Fe-doped β-Ga_2_O_3_ and other electronic properties from different theoretical approaches. Aside from the Fe dopant, intrinsic defects, including vacancies and interstitials, have significant impacts on their physical properties. However, the correlations among the Fe-doped β-Ga_2_O_3_ with native defects, the local crystal structure, and the electronic and optical properties have not been extensively studied.

Herein, we performed density functional theory (DFT) calculations to investigate the defect formation energies, charge transitional levels, electronic structures, and optical properties of Fe-doped β-Ga_2_O_3_, as well as Fe-doped β-Ga_2_O_3_ with different kinds of native defects. Our results address the controversies regarding the preferential Fe incorporation on the tetrahedrally or octahedrally coordinated Ga site, as mentioned above. Moreover, since the absence of relevant reports in Fe-doped β-Ga_2_O_3_ lattices with native defects from theoretical studies, the defect formation energies, charge transitional levels, and optical properties of the defective complexes of Fe-doped β-Ga_2_O_3_ with different kinds of native defects, i.e., oxygen vacancy (V_O_), gallium vacancy (V_Ga_), oxygen interstitial (O_i_), and gallium interstitial (Ga_i_), are investigated. Our studies are beneficial for understanding the ground state properties of Fe-doped β-Ga_2_O_3_, as well as for providing theoretical guidance on the design of β-Ga_2_O_3_-based functional materials and the promising applications of β-Ga_2_O_3_ for innovative spin-electronic and optoelectronic devices.

## 2. Calculation Methods

### 2.1. Computational Details

To implement the first-principles calculations, we use the Vienna ab initio Simulation Package (VASP) [27,28] based on DFT [29] with projected augmented wave (PAW) potentials. To characterize the exchange-correlation interactions, the generalized gradient approximation (GGA) parameterized by Perdew-Burke-Ernzerhof (PBE) [30] is used. The kinetic energy cutoff for the plane-wave basis set is 450 eV, the energy convergence criterion for the calculations is set to 1 × 10^−5^ eV/atom for the interactions between the electrons and ions, and all the atomic positions are fully optimized. When all components of the residual forces are less than 0.01 eV/Å, the relaxation will be terminated. A 4 × 4 × 2 Monkhost-Pack grid is utilized for structural relaxation, whereas a 9 × 9 × 4 Monkhost-Pack grid is used for the calculations of density of states (DOS) and optical properties. The so-called density function perturbation (DFPT) calculated method for phonon calculations is adopted in this work. Usually, phonon dispersion is needed to expand the supercell. However, we do not enlarge the supercell in this work considering the time-consuming nature, which may not influence our conclusions qualitatively. A 2 × 4 × 2 Monkhost-Pack grid and a 1 × 10^−6^ eV/atom energy criterion have been used for the calculation of phonon dispersion and mechanical properties. The valence electronic configurations for Ga, O, and Fe are [Ar] 3d^10^4s^2^4p^1^, [He] 2s^2^2p^4^, and [Ar] 3d^7^4s^1^, respectively.

**Figure 1:**
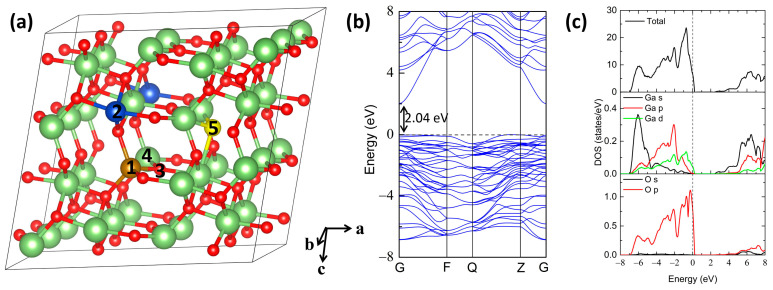
(**a**) The calculated complex model of a Fe-doped β-Ga_2_O_3_ supercell with native defects obtained from VESTA [35]. 1 and 2 denote the tetrahedrally and octahedrally coordinated Ga sites substituted by an iron atom, respectively. 3 and 4 show the vacancy sites for Ga and O, respectively, while 5 represents the interstitial positions for both Ga and O. The a, b, and c axes refer to the crystallographic a, b, and c directions, respectively. (For interpretation of the references to color in this figure legend, the reader is referred to the web version of this article.) (**b**,**c**) exhibit the band structure and the density of states for perfect β-Ga_2_O_3_, respectively.

A 1 × 2 × 2 β-Ga_2_O_3_ supercell of 32 Ga atoms and 48 O atoms is modeled in this study, with one Fe impurity replacing the Ga atom, corresponding to a doping concentration of 3.125%, as shown in Figure 1a. β-Ga_2_O_3_ possesses two inequivalent Ga positions. Fe impurity incorporation on the tetrahedrally and octahedrally coordinated Ga sites is labeled 1 and 2, respectively. Different kinds of native defects in terms of oxygen vacancy, gallium vacancy, oxygen interstitial, and gallium interstitial in the β-Ga_2_O_3_ supercell are considered, which are denoted as V_O_, V_Ga_, O_i_, and Ga_i_, respectively. For the atomic positions of V_O_ and V_Ga_, we use the results by Dong et al. [31], i.e., the positions of 3 and 4 in Figure 1a, respectively. For the low-energy O_i_ and Ga_i_ doping sites in gallium oxide, we adopt the results given by Zacherle et al. [32], where the two interstitial sites are located at the same position (0.683, 0.500, 0.459) in the supercell before relaxation and labeled as 5 in Figure 1a. For simplicity, Fe impurities replacing tetrahedral and octahedral Ga atoms are named Fe_GaI_ and Fe_GaΠ_, respectively. Thus, their complexes of Fe_GaI_ with V_O_, V_Ga_, O_i_, and Ga_i_ configurations are named Fe_GaI_V_O_, Fe_GaI_V_Ga_, Fe_GaI_O_i_, and Fe_GaI_Ga_i_, respectively, while complexes of Fe_GaΠ_ with V_O_, V_Ga_, O_i_, and Ga_i_ configurations are labeled as Fe_GaΠ_V_O_, Fe_GaΠ_V_Ga_, Fe_GaΠ_O_i_, and Fe_GaΠ_Ga_i_, respectively. Besides, we also employ the value of U in accordance with the experimental band gap for the perfect β-Ga_2_O_3_ [33], and the U value of 4.09 eV is adopted for the 3d orbital of the Fe dopant as suggested by the literature [34].

### 2.2. Formation Energies, Transitional Levels and Optical Calculations

The formation energy of the defect D in the charge state q is calculated as [36,37]

(1) $$H_{D,q}(E_{f},\mu )=[E_{D,q}-E_{p}]+\sum_{i}n_{i}\mu_{i}+q(E_{VBM}+E_{f})+E_{corr}$$

where $E_{D,q}$ and $E_{p}$ denote the total energy of the defect and perfect supercell, respectively. $n_{i}$ represents the number of i atoms added ($n_{i}<0$) or removed ($n_{i}>0$) from the perfect supercell, and $\mu_{i}$ is the corresponding chemical potential. $E_{VBM}$ is energy of the valence band maximum (VBM) for bulk Ga_2_O_3_. $E_{f}$ is Fermi level, which is referenced to the VBM in the bulk. $E_{corr}$ is the term that accounts for the finite-size corrections, which is determined by the potential alignment and is given as [36]

(2) $$E_{corr}=q(V_{D,q}^{r}-V_{p}^{r})$$

where the potential difference between the charged defect Ga_2_O_3_ supercell ($V_{D,q}^{r}$) and perfect Ga_2_O_3_ supercell ($V_{p}^{r}$) are calculated from the atomic-sphere-averaged potentials at the atomic sites farther away from the defect employed by the software of VASPKIT Standard Edition 1.3.5 [38].

Note that the chemical potential satisfies the boundary conditions as follows:

(3) $$2\mu_{Ga}+3\mu_{O}=\mu_{Ga_{2}O_{3}},\;\;\;\;\;\;\;\;\mu_{Ga}\leq \mu_{Ga}^{Metal},\;\;\;\;\;\;\;\;\;\;\;\mu_{O}\leq \frac{1}{2}\mu_{O_{2}}$$

Chemical potential varies according to different growth conditions. Under O-rich growth condition:

(4) $$\mu_{O}=\frac{1}{2}\mu_{O_{2}},\;\;\;\;\;\;\;\;\;\;\;\;\;\;\;\mu_{\mathrm{Ga}}=\frac{1}{2}(\mu_{Ga_{2}O_{3}}-\frac{3}{2}\mu_{O_{2}})$$

Under Ga-rich growth condition:

(5) $$\mu_{Ga}=\mu_{Ga}^{Metal},\;\;\;\;\;\;\;\;\;\;\;\;\;\;\;\mu_{O}=\frac{1}{3}(\mu_{Ga_{2}O_{3}}-2\mu_{Ga})$$

where, $\mu_{Ga_{2}O_{3}}$ is the chemical potential of the bulk β-Ga_2_O_3_. The chemical potential of $\mu_{Ga}^{Metal}$ and $\mu_{\mathrm{Fe}}$ are calculated from the energies of the most stable bulk crystal of the Ga and Fe atoms, respectively. $\mu_{O}$ represents the chemical potential of O obtained from the energy of O_2_. The chemical potentials of $\mu_{O}$, $\mu_{\mathrm{Ga}}$ and $\mu_{\mathrm{Fe}}$ under O-rich condition are −4.92 eV, −7.55 eV, −8.24 eV, respectively, while the corresponding values are −8.01 eV, −2.90 eV, −8.24 eV for Ga-rich atmosphere.

The transition energy $\varepsilon (q_{1}/q_{2})$ between charge state q_1_ and q_2_ for defect D doping configuration is calculated as [39]

(6) $$\varepsilon (q_{1}/q_{2})=\frac{E_{D}^{q_{1}}{|}_{E_{f}=0}-E_{D}^{q_{2}}{|}_{E_{f}=0}}{q_{2}-q_{1}}$$

Here, the $E_{D}^{q}{|}_{E_{f}=0}$ represents the formation energy of the defect D in charge state q evaluated at $E_{f}=0$. The $\varepsilon (q_{1}/q_{2})$ denotes the Fermi-level position where the charge states q_1_ and q_2_ have equal formation energy.

The absorption coefficients in optical properties can be described as [31,40]

(7) $$\alpha (\omega )=\sqrt{2}\omega {\left[\sqrt{\varepsilon_{1}^{2}(\omega )+\varepsilon_{2}^{2}(\omega )}-\varepsilon_{1}(\omega )\right]}^{\;1/2}$$

where $\varepsilon_{1}(\omega )$ and $\varepsilon_{2}(\omega )$ indicate the real and imaginary parts of the dielectric function, respectively. The $\varepsilon_{2}(\omega )$ can be calculated by summing up the transitions between occupied and unoccupied states using the following equation

(8) $$\varepsilon_{2}(\omega )=\left(\frac{4\pi^{2}e^{2}}{m\omega^{2}}\right){\sum_{i,j}\int \langle i\left|M\left|j\right.\right.\rangle }^{2}f_{i}(1-f_{i})\times \delta (E_{jk}-E_{ik}-\omega )d^{3}k$$

Here, m, e, M, and ω denote the mass of free electrons, the electron charge, the dipole matrix, and the frequency of incident photons, respectively. i, j, $f_{i}$, and k represent the initial state, the final state, the Fermi distribution function, and the wave function vector, respectively. The $\varepsilon_{2}(\omega )$ is related to the absorption of light and dielectric loss of energy, while $\varepsilon_{1}(\omega )$ is associated with the stored energy.

The energy loss function (ELF) can be described by the following equation [41]

(9) L(ω)=Im[−1ε(ω)]=ε2(ω)ε12(ω)+ε22(ω)

## 3. Results and Discussions

### 3.1. Structural Stability

The calculated lattice parameters of perfect β-Ga_2_O_3_ are a = 12.412 Å, b = 3.076 Å, c = 5.872 Å, and the unique angle β = 103.702°, which are in excellent accordance with the theoretically calculated values obtained by PBE [41] and B3PW [42] approaches, as well as with the experimental values [43], as shown in Table 1. The optimized structural parameters for the Fe-doped cases are also summarized in Table 1. The lattice constants of Fe-doped β-Ga_2_O_3_ exhibit a slight decrease, which can be ascribed to the comparable ionic radii and local structures between Fe and Ga atoms. The relative difference of the radii between Fe^3+^ (Fe^2+^) and Ga^3+^ ions is −1.61% (−11.3%). Fe_GaΠ_ is endowed with smaller lattice parameter variations in terms of all three lattice vectors and the unique angle β compared with Fe_GaI_, implying that small distortions may be easily formed in the experimental growth.

**Figure 2:**
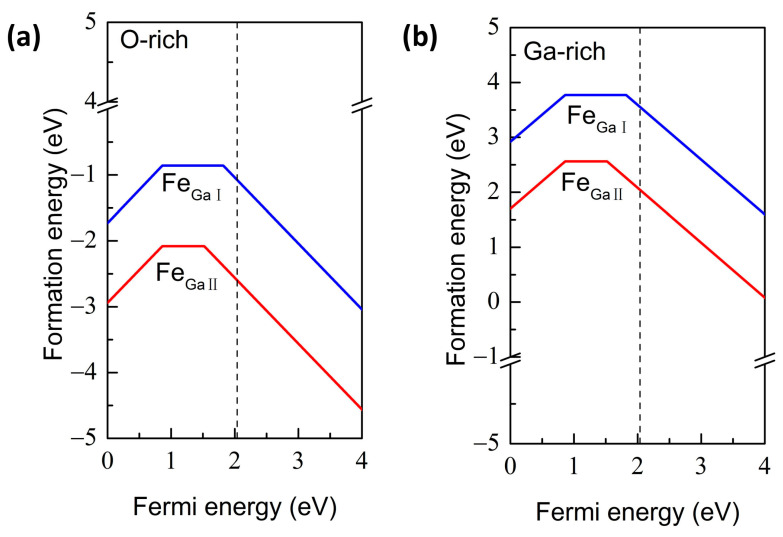
The defect formation energies of Fe-doped β-Ga_2_O_3_ under (**a**) the O-rich and (**b**) Ga-rich conditions. The dash line represents the calculated band gap of perfect β-Ga_2_O_3_.

To study the structural stability of Fe-doped β-Ga_2_O_3_ supercells, the defect formation energies under different conditions are calculated, as shown in Figure 2. Meanwhile, the transition levels are also employed to assess the ionization energies and the effectiveness of the doped systems. Our calculated value of the band gap for perfect β-Ga_2_O_3_ is 2.04 eV, as denoted by the dashed line in Figure 1b, which is consistent with the values obtained by the DFT calculated method but smaller than the experimental values [44]. The underestimated band gap for DFT calculation is a common phenomenon; however, it does not affect our conclusions qualitatively [45,46]. In addition, Figure 1c illustrates the calculated total density of states (TDOS) and partial density of states (PDOS) for perfect β-Ga_2_O_3_; the VBM is predominantly composed of O 2p orbital-derived states with minor hybridization with Ga 3d and 4p orbitals, while the CBM is mainly formed by Ga 4s orbitals.

Figure 2a indicates the formation energies for Fe-doped β-Ga_2_O_3_ under O-rich conditions. Fe_GaΠ_ case has lower formation energy, suggesting the preferential occupation of Fe_Ga_ at the octahedrally coordinated Ga site, which is in agreement with Ingebrigtsen’s conclusion by theoretical calculation [25] and other experimental results [19,20]. The thermodynamic transition level Fe^3+^/Fe^2+^ for Fe_GaΠ_, i.e., ε(0/−), is located at 0.52 eV below CBM, which is comparable to the theoretical value calculated by HSE hybrid functions (0.61 eV or 0.40 eV [25]), but slightly smaller than the reported experimental values (0.78 eV [25], 0.80 eV [26], 0.84 eV [17], 1.2 eV [22]). The transition level Fe^3+^/Fe^2+^ for Fe_GaI_ measured from VBM is 0.24 eV below CBM, thus deep acceptors are expected for both Fe-doped β-Ga_2_O_3_ configurations in the n-type β-Ga_2_O_3_ conditions, which can compensate for the free electrons caused by native defects or extrinsic impurities. In addition, the transition level ε(+/0) for both Fe_GaI_ and Fe_GaΠ_ is 0.86 eV above the VBM, which demonstrates that both doping cases generate deep donors even in the p-type β-Ga_2_O_3_ crystals. For the Ga-rich condition, i.e., the O-poor condition, as shown in Figure 2b, the tendency is the same as for the O-rich atmosphere, with the exception of higher formation energies. This suggests that Fe impurity is more easily substituted for Ga sites under O-rich conditions.

The mechanical characteristics of β-Ga_2_O_3_ are evaluated by employing a complete set of elastic constants. There are thirteen independent elastic constants (C_11_, C_22_, C_33_, C_44_, C_55_, C_66_, C_12_, C_13_, C_23_, C_15_, C_25_, C_35_, and C_46_) in the monoclinic symmetry crystal. The mechanical stability criteria of β-Ga_2_O_3_ are described as follows [47]:

(10) $$C_{ii}>0,\;\;\;i=1-6.$$

(11) $$[C_{11}+C_{22}+C_{33}+2(C_{12}+C_{13}+C_{23})]>0$$

(12) $$(C_{33}C_{55}-C_{35}^{2})>0$$

(13) $$(C_{44}C_{66}-C_{46}^{2})>0$$

(14) $$(C_{22}+C_{33}-2C_{23})>0$$

(15) $$[C_{22}(C_{33}C_{55}-C_{35}^{2})+2C_{23}C_{25}C_{35}-C_{23}^{2}C_{55}-C_{25}^{2}C_{33}]>0$$

(16) $$\begin{array}{ll} \Omega & =2[C_{15}C_{25}(C_{33}C_{12}-C_{13}C_{23})+C_{15}C_{35}(C_{22}C_{13}-C_{12}C_{23})\\ & +C_{25}C_{35}(C_{11}C_{23}-C_{12}C_{13})]-[C_{15}^{2}(C_{22}C_{33}-C_{23}^{2})\\ & +C_{25}^{2}(C_{11}C_{33}-C_{13}^{2})+C_{35}^{2}(C_{11}C_{22}-C_{12}^{2})]+C_{55}g>0 \end{array}$$

(17) $$g=C_{11}C_{22}C_{33}-C_{11}C_{23}^{2}-C_{22}C_{13}^{2}-C_{33}C_{12}^{2}+2C_{12}C_{13}C_{23}$$

In this study, the mechanical property calculations are carried out for the perfect and energetically favorable Fe_GaΠ_ doping configuration. The calculated elastic stiffness constants of prefect and Fe-doped β-Ga_2_O_3_ are shown in Table 2. For comparisons, available theoretical and experimental results are also listed. The elastic stiffness of prefect and Fe-doped β-Ga_2_O_3_ meets the mechanical stability criteria presented above, suggesting that prefect and Fe-doped β-Ga_2_O_3_ are mechanically stable at ambient conditions.

**Figure 3:**
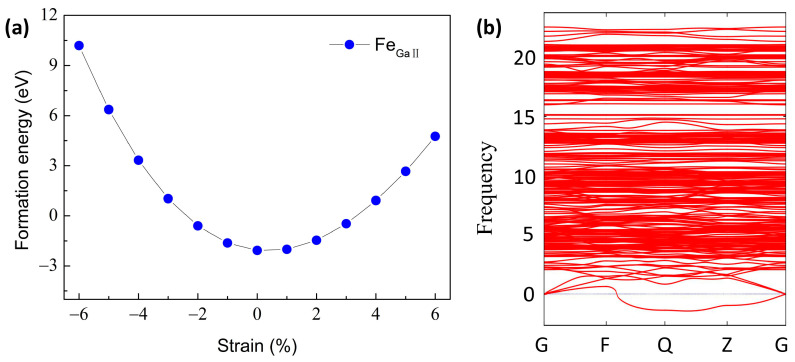
(**a**) The formation energies of Fe_GaΠ_ as a function of the biaxial strain with q = 0 under O-rich conditions. (**b**) The phonon dispersion calculations for the Fe_GaΠ_ doping configuration.

The formation energies as a function of the biaxial strain with q = 0 under O-rich conditions are shown in Figure 3a to assess the mechanical stability of Fe_GaΠ_ as well. It can be seen that the unstrained Fe_GaΠ_ is endowed with the smallest formation energy of −2.07 eV, indicating the Fe_GaΠ_ structure is in a stable state. Moreover, the formation energies are strongly dependent on the biaxial strain. As the tensile or compressive stress increases, it increases dramatically. When the compressive strain is greater than 2% or the tensile strain is higher than 3%, the defect formation energy is greater than 0, denoting that it may be difficult to materialize in the experiment. The defect formation energy increases more rapidly under compressive strain, which indicates that the defect is more difficult to realize under compressive strain.

Phonon analysis has proven to be an effective approach to predicting structural stability [51]. The phonon dispersion calculation for Fe_GaΠ_ doping structure is shown in Figure 3b. We observe three small imaginary frequencies, i.e., 0.64, 0.78, and 1.10 cm^−1^, locating at the non-gamma point (G). In general, imaginary frequencies at the gamma point can be related to structural instability, whereas the presence of imaginary frequencies at the non-gamma point can be responsible for the finite size of the simulation crystal cell, which can be eliminated by expanding the calculated supercell. Therefore, the Fe-doped β-Ga_2_O_3_ with small imaginary frequencies at the non-gamma point is predicted to be structurally stable, which agrees well with the results as suggested by the low formation energies.

**Figure 4:**
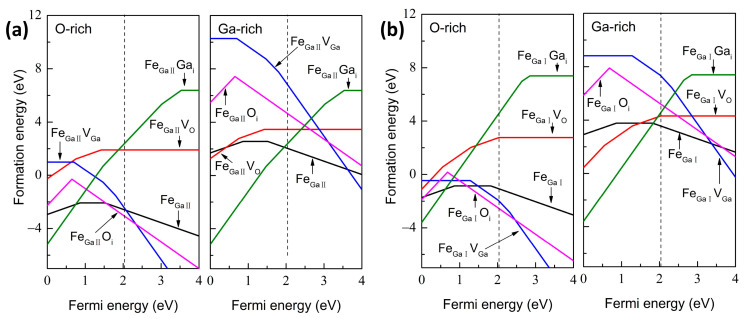
The defect formation energies under the O-rich and Ga-rich conditions for the complexes of (**a**) Fe_GaΠ_ and (**b**) Fe_GaI_ with native defects. The dash line represents the calculated band gap of perfect β-Ga_2_O_3_.

Figure 4a shows the complexes of Fe_GaΠ_ and Fe_GaI_ with native defects under different growth condition limits. For these defective Fe_GaΠ_ complexes under O-rich conditions, positively charged Fe_GaΠ_Ga_i_ and negatively charged Fe_GaΠ_O_i_ are energetically favorable when the E_f_ approaches the VBM and CBM, respectively, while positively charged Fe_GaΠ_Ga_i_ is expected throughout the whole band gap under Ga-rich condition. Moreover, the formation energies of Fe_GaΠ_Ga_i_ (Ga-rich condition) and Fe_GaΠ_O_i_ (O-rich condition) are lower compared with those of Fe_GaΠ_ case, suggesting that Fe_GaΠ_Ga_i_ and Fe_GaΠ_O_i_ complexes are easily formed under Ga-rich and O-rich conditions during experimental growth, respectively. For the Fe_GaΠ_V_O_ case, under O-rich conditions, the transition levels ε(+2/+1) and ε(+1/0) are 1.29 and 0.62 eV below CBM, respectively, indicating that the complex acts as a deep donor and cannot contribute to n-type conductivity. Different from the intrinsic Vo defect investigated in literature [10], we observe the +1 charge state rather than +2 and 0 charge states, which may be attributed to the combination of −1 charge state Fe dopant and +2 charge state V_O_. The similar results are inspected under Ga-rich conditions except for the lower formation energies, suggesting the Fe_GaΠ_V_O_ complex is easily formed in the O-poor growth atmosphere. For the Fe_GaΠ_V_Ga_ case, the transition levels ε(0/−2), ε(−2/−3) and ε(−3/−4) are located at 1.34, 0.57, and 2.04 eV below CBM, which demonstrate that the complexes act as deep acceptors with −4 or −3 charge state, respectively. Compared with the Ga-rich condition, the Fe_GaΠ_V_Ga_ complex possesses lower formation energies in n-type Ga_2_O_3_ materials, which demonstrates that the V_Ga_ is more likely produced in Fe-doped Ga_2_O_3_ under an O-rich growth atmosphere in experiments. It is in excellent agreement with the reported experimental result by Hany et al. [24]. Either under O-rich or Ga-rich conditions, positively charged and negatively charged Fe_GaΠ_O_i_ is energetically favorable when the Fermi level approaches the VBM and CBM, respectively. Moreover, Fe_GaΠ_O_i_ complex is more susceptible to being produced under O-rich conditions. For the Fe_GaΠ_Ga_i_ case, both under O-rich and Ga-rich conditions, positively charged are energetically favorable when the E_f_ is located throughout the whole band gap, which demonstrates that the complex exhibits n-type conductivity. The transition levels ε(+4/+3) and ε(+3/+2) are 1.47 and 3.02 eV above the VBM under O-rich condition and Ga-rich condition, which indicates that the +3 charge state for the Fe_GaΠ_Ga_i_ complex is expected. In addition, the higher formation energies under the O-rich condition for the Fe_GaΠ_Ga_i_ illustrate that the complex is more likely to be found under the Ga-rich conditions. It is worth mentioning that the Ga_i_ is the main origin of the native defect to form the n-type conductive β-Ga_2_O_3_ crystal, as illustrated in Refs. [8,9], while a low formation energy is gained for the Fe_GaΠ_Ga_i_ complex. The E_f_ always tends to be positioned at the higher region of the bandgap in β-Ga_2_O_3_ and gives rise to the n-type conduction characteristic due to unintentionally introduced native defects during the growth of β-Ga_2_O_3_. Therefore, our calculated results illustrate that the +3 charge state Fe_GaΠ_Ga_i_ under O-poor condition and −2 charge state Fe_GaΠ_O_i_ under O-rich condition are easily formed for the growth of β-Ga_2_O_3_ crystals.

Figure 4b shows the defect formation energies for the complexes of Fe_GaI_ with native defects. Different from the case of Fe_GaΠ_ complexes, under O-rich conditions, +4 charge state Fe_GaI_Ga_i_ and −2 charge state Fe_GaI_O_i_ are dominated when the E_f_ is located near the VBM and CBM, respectively, while −4 charge state Fe_GaI_Ga_i_ is easily generated throughout the whole band gap under Ga-rich conditions. Thus, different local structures can influence the electron transfer. As shown in Figure 4b, these defective complexes are characterized by similar tendencies with those of Fe_GaΠ_ complexes in exception for different formation energies both under O-rich and Ga-rich conditions.

### 3.2. Optical Property

**Figure 5:**
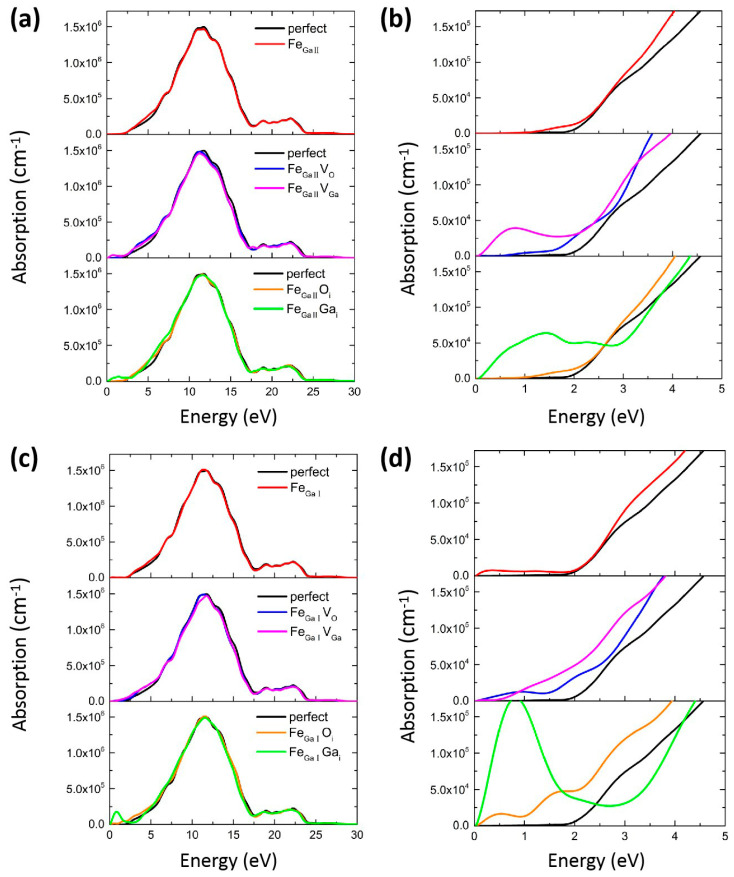
Comparison of the optical absorption spectra of perfect, Fe-doped, as well as various Fe_GaΠ_ and Fe_GaI_ complexes in energy range from 0–30 eV (**a**,**c**). Panels (**b**,**d**) show the corresponding amplified spectra at the low-energy region (0–5 eV).

For wide-band gap semiconductor materials, optical parameters of dielectric function ε(ω) can be employed to clarify the linear response of the system to electromagnetic radiation, which is crucial to assessing the interactions between photons and electrons. The imaginary part $\varepsilon_{2}(\omega )$ of the dielectric constant is related to the absorption of light and the dielectric energy-loss function, while $\varepsilon_{1}(\omega )$ is associated with the stored energy. Figure 5 denotes the optical absorption coefficient of perfect, Fe-doped, and various Fe_GaΠ_/Fe_GaI_ complexes in the energy range between 0 and 30 eV. Figure 5b,d exhibits the enlarged plots at the (0–5) eV region. The strong absorption peaks are located at 11.8 and 10.9 eV for perfect β-Ga_2_O_3_, as shown in Figure 5a, which originate from the inter-band transitions from O 2p states to Ga 4s states, illustrating that the bulk material is characterized by its deep ultraviolet properties. The calculated data is consistent with Yan and Pan’s results [52,53]. Compared with perfect β-Ga_2_O_3_, the profiles of Fe-doped β-Ga_2_O_3_ and various complexes in Figure 5a,c are endowed with similar absorption peaks in the high-energy ultraviolet region, indicating that these dopants can hardly decrease the optical absorption coefficients of β-Ga_2_O_3_ in the deep ultraviolet region. The slightly red shift for Fe_GaΠ_V_Ga_, Fe_GaI_V_Ga_, Fe_GaΠ_O_i_, and Fe_GaI_O_i_ can be ascribed to hole doping, while the blue shift for the Fe_GaΠ_Ga_i_, Fe_GaI_Ga_i_ cases can be associated with the introduction of electrons, which is consistent with our formation energy calculations above. In addition, one can obviously notice that new peaks appear in the low-energy region for these Fe_GaΠ_/Fe_GaI_ complexes, as shown in the amplified plots shown in Figure 5b,d.

The perfect β-Ga_2_O_3_ possesses an optical band gap of about 2 eV, which is in good agreement with the value observed from electronic structure calculations in Figure 1b. For the Fe_GaΠ_ and Fe_GaI_ cases, the optical absorption spectra remain almost unchanged in the visible region, which can be associated with the deep acceptor doping for the Fe foreigner atom. When introducing extra V_O_ into the β-Ga_2_O_3_ crystal, the absorption coefficients become relatively low for both Fe_GaΠ_V_O_ and Fe_GaI_V_O_ cases in the low-energy region (0–5 eV), whereas a new wide peak for Fe_GaΠ_V_Ga_ configuration is generated, leading to the optical migration from the ultraviolet light region to the visible-infrared region. The new peak for Fe_GaΠ_V_Ga_ configuration originated from the inter-band transitions of O 2p from the VBM to the induced impurity levels. Similarly, a new peak appears at a high energy level of ~1.42 eV and ~0.84 eV for Fe_GaΠ_Ga_i_ and Fe_GaI_Ga_i_ complexes, respectively, which are originated by the transitions from impurity levels to Ga 4s orbitals. These new peaks are expected to benefit the optical transformation from ultraviolet light to the visible-infrared region. In the low-energy region in Figure 5b,d, the Fe_GaΠ_O_i_ case exhibits the absence of a clear optical absorption peak, while a few small oscillation peaks are present for the Fe_GaI_O_i_ combination. Therefore, Fe_GaΠ_Ga_i_, Fe_GaI_Ga_i_, and Fe_GaΠ_V_Ga_ complexes can significantly enhance the optical absorption in the visible-infrared region.

**Figure 6:**
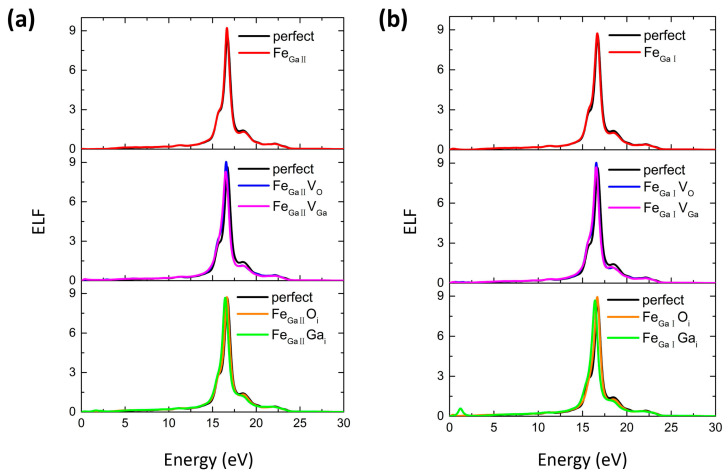
The energy-loss function (ELF) for (**a**) Fe_GaΠ_ and (**b**) Fe_GaI_ complexes.

The energy-loss function (ELF) is calculated based on Equation (9) from the dynamic dielectric constant at small scattering angles, which can determine the energy loss of free electrons across the material. This ELF function allows a direct comparison between theoretical conclusions and experimental spectroscopy measurements such as EELS [54]. Figure 6 shows the ELF spectra for Fe_GaΠ_ and Fe_GaI_ complexes. The major peak for perfect β-Ga_2_O_3_ is located at 16.7 eV. The peak positions remain at the same locations for Fe_GaΠ_ and Fe_GaI_, while accompanying with higher ELF value. This indicates that the induced Fe dopant tends to increase its energy loss and decreases its emission of peak energy efficiency under the high energy region in the material. The changes in the primary peaks for these Fe_GaΠ_ and Fe_GaI_ complexes are also minor, showing that the energy loss in the β-Ga_2_O_3_ material is almost negligible after the extra introduction of various native defects. Additionally, a seemingly little peak develops in the low-energy region for the Fe_GaI_Ga_i_ case, which may be attributed to the optical absorption peak of ~0.84 eV in Figure 5d.

### 3.3. GGA + U

We further carried out the GGA + U calculations to gain insight into the influences on the electronic structure of Fe-doped β-Ga_2_O_3_, considering the strong electron-electron interactions. GGA + U calculations are usually applied to deal with strong correlations in localized d- or f- electron systems and thus partially solve the band gap underestimation. We employ the values of U in the literature to accord with the experimental band-gap for the perfect β-Ga_2_O_3_ [33]. Besides, the U value of 4.09 eV has been chosen for the 3d orbital of the Fe dopant based on the literature [34].

**Figure 7:**
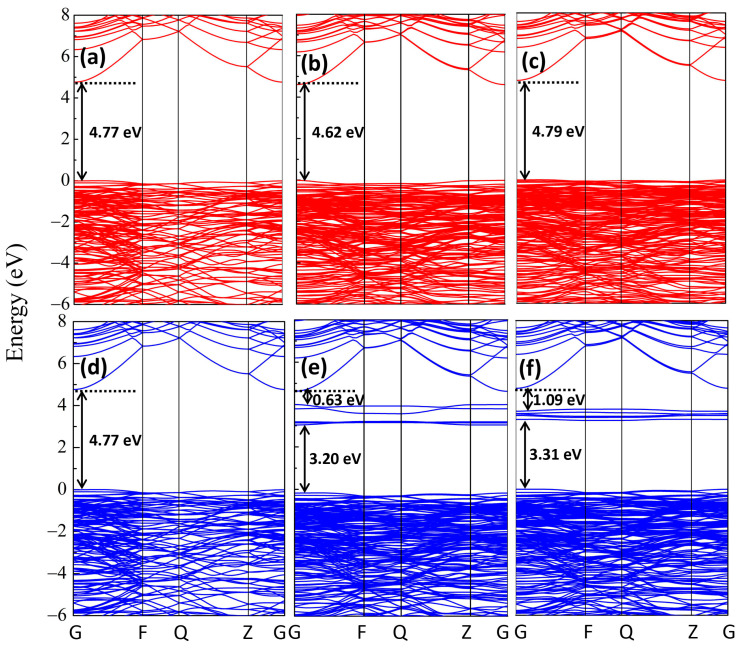
GGA + U calculated the band structures of (**a**) spin-up channel and (**d**) spin-down channel for perfect β-Ga_2_O_3_, (**b**) spin-up channel and (**e**) spin-down channel for Fe_GaΠ_ structure, as well as (**c**) spin-up channel and (**f**) spin-down channel for perfect Fe_GaI_ structure.

The GGA + U calculated spin-up and spin-down band structures of the perfect β-Ga_2_O_3_ configuration are shown in Figure 7a,d. The perfect β-Ga_2_O_3_ is endowed with a band gap of 4.77 eV accompanied by a direct semiconductor structure, which is in good agreement with the experimental band-gap value of 4.78 eV [55]. The much flat valence bands illustrate a large effective mass and low mobility, which prevent the formation of p-type β-Ga_2_O_3_. The main orbital compositions of the VBM and CBM are consistent with those from GGA calculations, as mentioned before. Figure 7b,c,e,f show the spin-up and spin-down band structures of Fe_GaΠ_ and Fe_GaI_ structures, respectively, where the Fe impurity bands of 3d characters are located above the E_f_ near CBM. The Fe_GaΠ_ and Fe_GaI_ exhibit semiconductor ferromagnetic ground states. The magnetic moment mainly originates from the uncompensated spin-down orbitals of Fe impurities, where no extra band has been observed in the spin-up channel. In the spin-down channel, several isolated bands originated from Fe 3d orbitals are located above the E_f_, which exhibit deep acceptor levels and give rise to a magnetic moment of 5 μ_B_. The induced deep acceptor levels of Fe 3d orbitals are 0.63 eV lower than the CBM and 3.20 eV higher than the VBM in the Fe_GaΠ_ structure. For the Fe_GaI_ configuration, in the spin-down channel, several isolated bands originated from Fe 3d orbitals are located at 3.31 eV, 3.49 eV, 3.57 eV, 3.59 eV, and 3.70 eV above the E_f_. The induced deep acceptor levels of Fe 3d orbitals are 1.09 eV lower than the CBM. The larger values below CBM compared with these of GGA calculations can be attributed to the more localized Ga and Fe 3d orbitals under GGA + U calculations, which are reasonable compared with the experimentally measured results, i.e., the EPR results of 0.84 eV observed from Polyakov et al. [26] and 1.2 eV from Bhandari et al. [22].

### 3.4. Conclusions

Based on first-principle DFT calculations with the GGA approach, we illustrate that Fe dopant is energetically favored for the octahedrally coordinated Ga site in Fe-doped Ga_2_O_3_ material. The controversy regarding the preferential Fe incorporation on the Ga site in the β-Ga_2_O_3_ crystal has been addressed; our result demonstrates that Fe dopant is energetically favored for the octahedrally coordinated Ga site, while analyses based on phonon dispersion mechanical characteristics, strain-dependent analyses, and formation energies are used to confirm the structural stability. Moreover, Fe impurities are more easily substituted for Ga sites under O-rich conditions. Our calculated results illustrate that the +3 charge state Fe_GaΠ_Ga_i_ under O-poor conditions and −2 charge state Fe_GaΠ_O_i_ under O-rich conditions are easily formed for the growth of β-Ga_2_O_3_ crystals. The formation energy calculations predict that the V_Ga_ and O_i_ in Fe-doped Ga_2_O_3_ are more likely to be formed under an O-rich growth environment. When the E_f_ approaches the valence and conduction band edges, the +3 charge state Fe_Ga_Ga_i_ and −2 charge state Fe_Ga_O_i_ are energetically advantageous, respectively. Moreover, V_O_ and Ga_i_ are expected under Ga-rich conditions with the preferred −4 charge sate Fe_Ga_Ga_i_ complex throughout the whole band gap. Fe_GaΠ_Ga_i_, Fe_GaI_Ga_i_, and Fe_GaΠ_V_Ga_ complexes can significantly enhance the optical absorption in the visible-infrared region. The changes in the primary peaks for these Fe_GaΠ_ and Fe_GaI_ complexes are all minor, showing that the energy loss in the β-Ga_2_O_3_ material is almost negligible after the introduction of various native defects. The GGA + U calculations show that the induced deep acceptor levels of Fe 3d orbitals are 0.63 eV and 1.09 eV lower than the CBM for Fe_GaΠ_ and Fe_GaI_ configurations, respectively, which are reasonable compared with the experimentally measured results, i.e., the EPR results of 0.84 eV observed from Polyakov et al. [26] and 1.2 eV from Bhandari et al. [22].

## Figures and Tables

**Table 1:** The calculated lattice constants for perfect and Fe-doped β-Ga_2_O_3_. The values in parentheses indicate the changes in lattice parameters compared with these of the perfect β-Ga_2_O_3_.

Lattice Constants	Perfect (This Work)	Perfect (Literature)	Fe_GaI_	Fe_GaΠ_
a (Å)	12.412	12.494 [41]/12.28 [42]/12.214 [43]	12.387 (−0.20%)	12.393 (−0.15%)
b (Å)	3.076	3.096 [41]/3.05 [42]/3.037 [43]	3.076 (0.02%)	3.075 (−0.05%)
c (Å)	5.872	5.898 [41]/5.82 [42]/5.798 [43]	5.888 (0.28%)	5.868 (−0.07%)
β (°)	103.702	103.705 [41]/103.83 [43]	103.835 (0.13%)	103.700 (0%)

**Table 2:** Calculated elastic coefficients C_ij_, bulk modulus B_H_, Yong modulus E_H_ and shear modulus G_H_ (all in GPa) for perfect and Fe_GaΠ_ structures, as well as the literature values for comparison. The subscript H is responding to Voigt-Reuss-Hill notation.

		C_11_	C_12_	C_13_	C_15_	C_22_	C_23_	C_25_	C_33_
Perfect	This work (PBE)	215	109	119	–15	317	72	14	312
	PBE [48]	208	118	146	0	335	83	0	318
	B3LYP [49]	235	124	138	−13	357	76	7	357
	Exp [50]	238	130	152	−4	359	78	2	346
Fe_GaΠ_		217	107	118	−17	322	72	9	315
		C_35_	C_44_	C_46_	C_55_	C_66_	B_H_	E_H_	G_H_
Perfect	This work (PBE)	6	48	14	64	94	159	188	72
	PBE [48]	19	50	9	77	96	171	192	73
	B3LYP [49]	12	55	15	81	101	179	214	82
	Exp [50]	19	49	6	91	107	184	213	82
Fe_GaΠ_		7	51	18	67	90	160	190	73

## Data Availability

No new data were created or analyzed in this study. Data sharing is not applicable to this article.
